# Analysis of Outcomes in Pancreaticoduodenectomy for Elderly Patients Aged 80 and Above: A Retrospective Study Using Inverse Probability of Treatment Weighting

**DOI:** 10.1002/agm2.70048

**Published:** 2025-10-13

**Authors:** Tianxiao Wang, Yue Qiu, Yingjixing Luo, Ruili Wei, Li Xu, Jia Huang, Wenying Zhou, Hanchun Huang, Yongliang Sun, Zhiying Yang

**Affiliations:** ^1^ Peking University China‐Japan Friendship School of Clinical Medicine Beijing China; ^2^ China‐Japan Friendship Hospital (Institute of Clinical Medical Science) Chinese Academy of Medical Science & Peking Union Medical College Beijing China; ^3^ School of Clinical Medicine Beijing University of Chinese Medicine Beijing China; ^4^ Capital Medical University China‐Japan Friendship School of Clinical Medicine Beijing China; ^5^ Department of Hepatobiliary and Pancreatic Surgery China‐Japan Friendship Hospital Beijing China

**Keywords:** 80 and over, aged, outcomes, pancreaticoduodenectomy, statistical weighting

## Abstract

**Objectives:**

With an aging population, more elderly individuals are facing the decision to undergo complex surgeries, such as pancreaticoduodenectomy (PD). This study evaluates the safety and feasibility of PD in patients aged 80 and above.

**Methods:**

We conducted a retrospective analysis of 422 patients who underwent PD between August 2011 and January 2024. The patients were categorized into three age groups: < 60 years, 60–79 years, and ≥ 80 years. We adjusted for baseline characteristics using inverse probability of treatment weighting (IPTW) and compared postoperative complications, hospital stay durations, 90‐day mortality, and long‐term survival across the groups. A subgroup analysis identified factors associated with complications and 90‐day mortality.

**Results:**

After IPTW adjustment, no significant differences in complications or long‐term survival were observed between the groups. However, the ≥ 80‐year group had a significantly higher 90‐day mortality rate (13.64%) compared to the 60–79 years group (2.00%) and the < 60 years group (1.46%) (*p* = 0.002). Subgroup analysis identified hypertension, coronary artery disease (CAD), and nutritional risk (NRS ≥ 3) as independent risk factors for complications, while age ≥ 80 and CAD were associated with higher 90‐day mortality.

**Conclusions:**

Age does not significantly impact postoperative complications or long‐term survival following PD. While PD is safe and feasible for the elderly, those with cardiovascular comorbidities or poor nutritional status face higher risks of complications and mortality. A comprehensive preoperative assessment is critical to minimizing these risks.

## Graphical abstract

This graphic summary shows the main clinical outcomes of patients over 80 years of age following pancreaticoduodenectomy (PD) after adjusting for propensity score weighting (IPTW). Compared with younger patients, there was no significant difference in postoperative complication rates or overall survival time in elders.

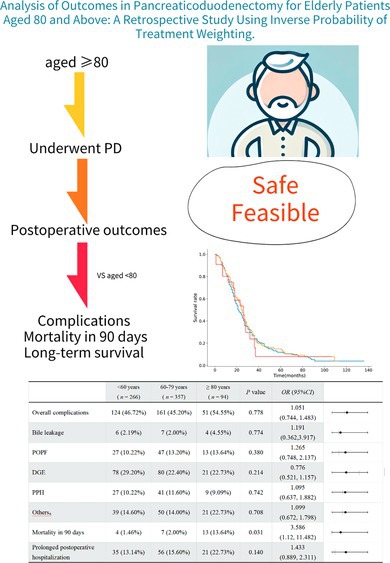

## 1 Introduction

The increasing global population of elderly individuals has led to a rise in the number of elderly patients undergoing complex surgical procedures, such as pancreaticoduodenectomy (PD) [1]. However, the suitability of PD for patients aged 80 and older remains debated due to concerns about higher risks of complications and mortality [2, 3].

Historically, PD has been less commonly performed in elderly patients, particularly those over 80, due to the complexity of the surgery and the extent of the resection involved [4, 5, 6, 7]. Although several studies have explored PD outcomes in elderly populations, the impact of advanced age, especially in octogenarians, on postoperative results is still unclear [8, 9]. This retrospective study aims to evaluate the safety and feasibility of PD in patients aged 80 and above by comparing postoperative complications, mortality, and long‐term survival.

## 2 Materials and Methods

### 2.1 Patient Selection and Data Collection

We retrospectively analyzed 422 patients who underwent PD at China‐Japan Friendship Hospital from August 2011 to January 2024. The patients were divided into three age groups: < 60 years, 60–79 years, and ≥ 80 years. Data collected included baseline characteristics such as age, sex, body mass index [BMI], pathological diagnosis, comorbidities (diabetes, hypertension, coronary artery disease [CAD], cerebrovascular disease [CVD], chronic kidney disease [CKD], history of chronic pancreatitis, alcohol consumption, other abdominal surgeries, and other malignant tumors), preoperative assessment such as nutrition risk screening 2002 (NRS 2002) [10], American Society of Anesthesiologists (ASA) grading scale [11], and the Charlson comorbidity Index (CCI) [12], and laboratory results including white blood cell count (WBC), hemoglobin (HGB), platelet count (PLT), serum total bilirubin (TB), serum albumin, serum creatinine (Scr), international normalized ratio (INR), and D‐dimer.

### 2.2 Preoperative Management

All patients underwent standard preoperative evaluations, including multi‐organ function tests and anesthesia assessments. Surgery was performed after stabilizing patients with dehydration, malnutrition, anemia, or coagulation abnormalities. For patients with obstructive jaundice, preoperatively biliary drainage techniques, including percutaneous transhepatic cholangiography and drainage (PTCD), percutaneous transhepatic biliary drainage (PTBD), endoscopic naso‐biliary drainage (ENBD), and biliary stent placement.

### 2.3 Surgical Technique

PD surgeries were categorized into open and laparoscopic approaches, further subdivided into classic PD and pylorus‐preserving pancreaticoduodenectomy (PPPD). In cases of vascular involvement, vascular resection was performed. The preferred technique for pancreatojejunostomy was an end‐to‐side anastomosis, with alternative techniques used as needed. The gastrointestinal anastomosis in classic PD was positioned either antecolically or retrocolically. Typically, three abdominal drainage tubes were inserted: one near the bile duct–jejunum anastomosis, and the other two near the pancreaticojejunostomy and splenic fossa.

### 2.4 Outcomes and Follow‐Up

Primary outcomes included postoperative complications such as bile leakage, postoperative pancreatic fistula (POPF), delayed gastric emptying (DGE), post‐pancreatectomy hemorrhage (PPH), and other complications (e.g., pneumonia, wound or abdominal infections, pleural or abdominal effusion, chylous leakage, acute myocardial infarction, and pulmonary embolism). The definitions of bile leakage, POPF, DGE, and PPH were based on the criteria established by the International Study Group of Pancreatic Surgery (ISGPS) and the International Study Group of Liver Surgery (ISGLS) [13, 14, 15, 16]. POPF includes grades B and C. Grade A of POPF, which was defined as a biochemical leak, was not included in the statistics [13]. Starting from the second postoperative day, any fluid containing bile that drains through the abdominal drainage tube, regardless of the volume, is considered bile leakage. Postoperative bleeding from the abdominal drainage tube or gastrointestinal bleeding, a hemoglobin decrease exceeding 30 g/L, or apparent signs of hypovolemia requiring transfusion of more than 3 units of red blood cells or invasive procedures (such as arterial embolization, endoscopic treatment, or reoperation), are considered PPH. DGE is diagnosed if, 10 days postoperatively, gastric decompression fluid remains greater than 300 mL/day, if the gastric tube needs to be reinserted due to vomiting after removal for feeding, or if oral intake is still not possible 14 days postoperatively despite the resolution of other complications, and upper gastrointestinal contrast imaging confirms gastric retention and impaired gastric motility. Other recorded complications included pneumonia, wound or abdominal cavity infections, pleural or abdominal effusions, chylous leakage, acute myocardial infarction, and pulmonary embolism. The complications were diagnosed by specialized physicians. Secondary outcomes included 90‐day postoperative mortality, prolonged postoperative hospitalization (> 30 days), and long‐term survival. Long‐term follow‐up was conducted every 3 months for at least 3 years or until the patient was lost to follow‐up or deceased.

### 2.5 Statistical Analysis

Continuous variables were presented as mean ± standard deviation, and categorical variables as counts and percentages. Group comparisons were made using analysis of variance (ANOVA) for continuous variables and chi‐square tests for categorical variables. A *p‐*value of less than 0.05 was considered statistically significant. Statistical analysis was conducted using Python 3.0 and Jupyter Notebook (Python Software Foundation, https://www.python.org/). Inverse probability of treatment weighting (IPTW) was used to adjust for baseline differences, with sensitivity analysis using propensity score matching (PSM). Logistic regression models were used to identify factors associated with postoperative complications and mortality.

## 3 Results

### 3.1 Overall Characteristics of Patients and Outcomes

**FIGURE 1:**
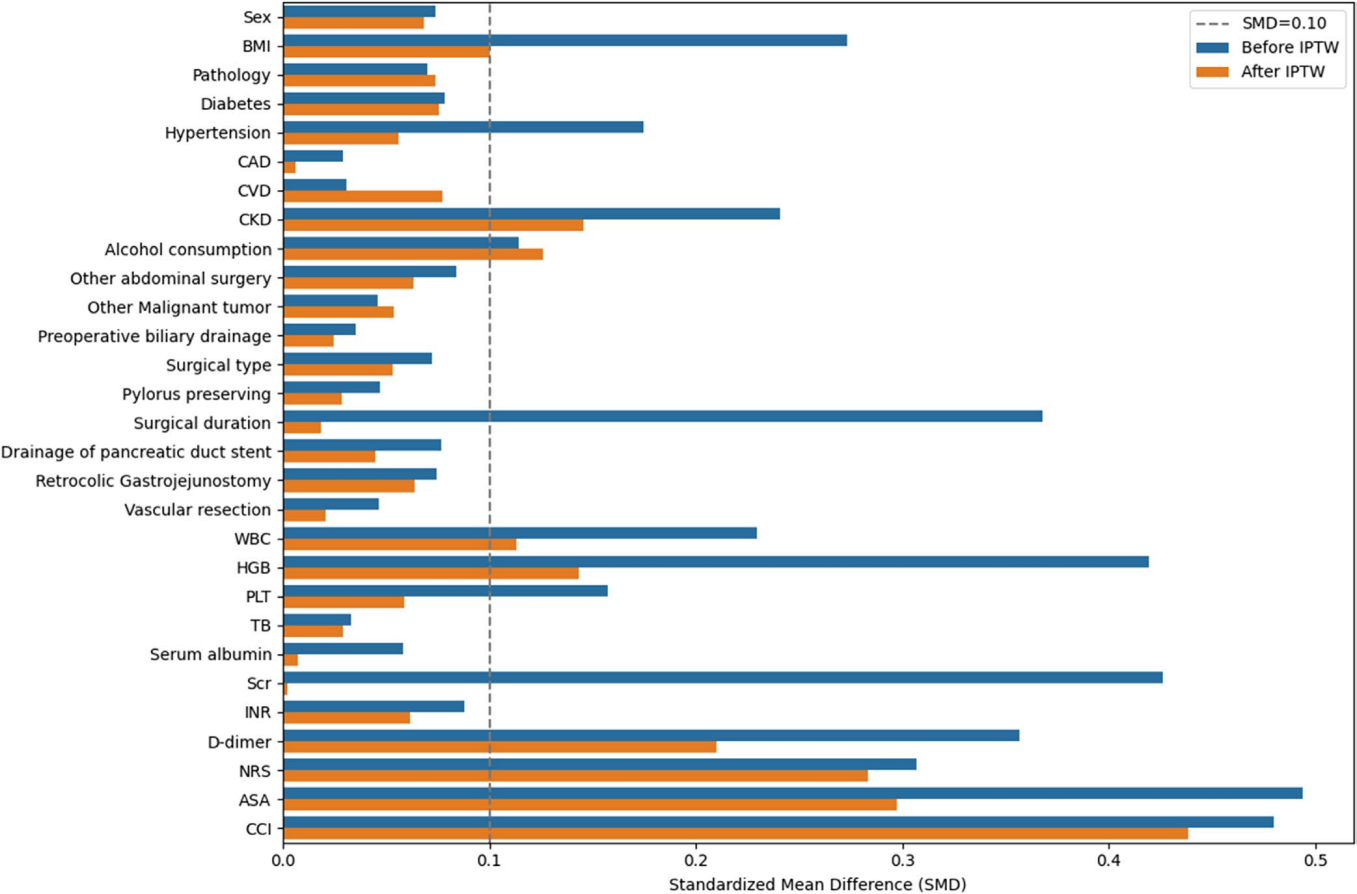
Comparison of Standardized Mean Difference (*SMD*) before and after IPTW. Blue bars represent before IPTW, orange bars represent after IPTW, and the gray dotted line represents the *SMD* value is 0.10.

Among the 422 patients included in the analysis, there were 139 patients in the age group of < 60 years, 260 patients in the age group of 60–79 years, and 23 patients in the age group of ≥ 80 years. The baseline demographic and clinical characteristics of the patients before and after applying IPTW are summarized in Table 1. Before IPTW, there were significant differences among the three groups in terms of BMI, history of hypertension and alcohol consumption, CKD, surgical duration, NRS, ASA, CCI, preoperative WBC, Scr, HGB, D—dimer, and PLT (*SMD* > 0.10). After IPTW, the differences among the groups for the vast majority of the baseline data were significantly controlled (*SMD* ≤ 0.10) except for CKD, history of alcohol consumption, WBC, HGB, D—dimer, NRS, ASA, and CCI (Figure 1).

**TABLE 1:** Clinical characteristics among patients < 60 years, 60–79 years and ≥ 80 years.

	Before IPTW				After IPTW			
	< 60 years (*n* = 139)	60–79 years (*n* = 260)	≥ 80 years (*n* = 23)	*SMD*	< 60 years (*n* = 266)	60–79 years (*n* = 357)	≥ 80 years (*n* = 94)	*SMD*
Sex (Male)	94 (67.63%)	169 (65.00%)	13 (56.52%)	0.074	179 (67.15%)	228 (64.00%)	51 (54.55%)	0.068
BMI (kg/m^2^)	24.03 ± 3.68	22.93 ± 3.19	23.41 ± 3.12	0.273	24.12 ± 3.67	22.98 ± 3.20	23.55 ± 3.19	0.100
Pathology								
Bile duct cancer	16 (11.51%)	63 (24.23%)	6 (26.09%)		31 (11.68%)	84 (23.60%)	26 (27.27%)	
Pancreatic cancer	49 (35.25%)	86 (33.08%)	9 (39.13%)	0.070	93 (35.04%)	116 (32.40%)	34 (36.36%)	0.074
Periampullary cancer	37 (26.62%)	77 (29.62%)	5 (21.74%)		72 (27.01%)	108 (30.40%)	21 (22.73%)	
Other benign diseases	37 (26.62%)	34 (13.08%)	3 (13.04%)		70 (26.28%)	49 (13.60%)	13 (13.64%)	
Diabetes	19 (13.67%)	66 (25.83%)	5 (21.74%)	0.078	35 (13.14%)	89 (24.80%)	21 (22.73%)	0.076
Hypertension	30 (21.58%)	100 (38.46%)	11 (47.83%)	0.174	56 (21.17%)	134 (37.60%)	47 (50.00%)	0.056
CAD	6 (4.32%)	14 (5.38%)	2 (8.70%)	0.029	12 (4.38%)	19 (5.20%)	9 (9.09%)	0.006
CVD	0 (0.00%)	12 (4.62%)	1 (4.35%)	0.030	0 (0.00%)	14 (4.00%)	4 (4.55%)	0.077
CKD								
1	86 (61.87%)	73 (28.08%)	1 (4.35%)		167 (62.77%)	99 (27.60%)	4 (4.55%)	
2	44 (31.65%)	129 (49.62%)	6 (26.09%)	0.240	85 (32.12%)	177 (49.60%)	26 (27.27%)	0.146
3	8 (5.76%)	57 (21.92%)	15 (65.22%)		14 (5.11%)	80 (22.40%)	60 (63.64%)	
4	1 (0.72%)	1 (0.38%)	1 (4.35%)		0 (0.00%)	1 (0.40%)	4 (4.55%)	
Chronic pancreatitis	9 (6.47%)	10 (3.85%)	0 (0.00%)	0.043	17 (6.57%)	14 (4.00%)	0 (0.00%)	0.031
Alcohol consumption	42 (30.22%)	45 (17.31%)	3 (13.04%)	0.114	80 (29.93%)	61 (17.20%)	13 (13.64%)	0.126
Other abdominal surgery	20 (14.39%)	44 (16.92%)	1 (4.35%)	0.083	37 (13.87%)	59 (16.40%)	4 (4.55%)	0.063
Other malignant tumors	9 (6.47%)	18 (6.92%)	0 (0.00%)	0.046	17 (6.57%)	26 (7.20%)	0 (0.00%)	0.054
Preoperative biliary drainage	36 (25.90%)	81 (31.15%)	7 (30.43%)	0.035	66 (24.82%)	109 (30.40%)	26 (27.27%)	0.024
Surgery type								
Classic PD	119 (85.61%)	219 (84.23%)	21 (91.30%)		227 (85.40%)	300 (84.00%)	85 (90.91%)	0.029
PPPD	20 (14.39%)	41 (15.77%)	2 (8.70%)	0.047	39 (14.60%)	57 (16.00%)	9 (9.09%)	
Surgery type								
Open surgery	124 (89.21%)	245 (94.23%)	23 (100.00%)		237 (89.05%)	336 (94.00%)	94 (100.00%)	0.053
Laparoscopic surgery	15 (10.79%)	15 (5.77%)	0 (0.00%)	0.071	29 (10.95%)	21 (6.00%)	0 (0.00%)	
Surgical duration (min)	360 ± 98.40	390 ± 100.67	360 ± 96.71	0.368	360 ± 98.75	382.5 ± 100.83	367.5 ± 98.88	0.019
Intraoperative blood loss (ml)	400 ± 801.81	400 ± 521.01	400 ± 290.85	0.202	400 ± 808.60	400 ± 517.86	400 ± 288.11	0.278
Drainage of the pancreatic duct stent	127 (91.37%)	357 (89.4)	23 (100.0)	0.076	247 (92.70%)	318 (89.20%)	94 (100.00%)	0.045
Gastrojejunostomy								
Retrocolic	109 (78.42%)	210 (80.77%)	16 (69.57%)	0.074	208 (78.10%)	286 (80.00%)	68 (72.73%)	0.064
Antecolic	30 (21.58%)	50 (19.23%)	7 (30.43%)		58 (21.90%)	71 (20.00%)	26 (27.27%)	
Vascular resection	17 (12.23%)	27 (10.38%)	4 (17.39%)	0.046	33 (12.41%)	39 (10.80%)	17 (18.18%)	0.021
WBC (*10^9^/L)	6.14 ± 2.62	6.53 ± 3.00	7.15 ± 11.44	0.229	6.12 ± 2.60	6.49 ± 2.99	7.23 ± 11.65	0.113
HGB(g/L)	124 ± 18.35	121 ± 20.32	113 ± 27.08	0.419	124 ± 18.26	120.5 ± 20.28	113.5 ± 27.67	0.143
PLT (*10^9^/L)	231 ± 82.25	245 ± 86.31	208 ± 77.90	0.157	231 ± 79.67	244 ± 87.00	212 ± 77.51	0.059
TB (μmol/L)	35.56 ± 101.63	45.4 ± 99.44	48.2 ± 89.70	0.032	35.48 ± 101.16	45.4 ± 99.82	49.6 ± 91.03	0.029
Serum albumin	39 ± 19.67	39 ± 19.74	36 ± 32.56	0.058	39 ± 19.89	39 ± 20.03	36.2 ± 33.28	0.007
Scr (μmol/L))	61.4 ± 25.17	59.95 ± 16.89	79.95 ± 24.55	0.426	61.15 ± 15.85	59.95 ± 16.91	78.525 ± 23.31	0.002
PTA(s)	103 ± 13.93	105.25 ± 18.66	98 ± 17.24	0.194	103 ± 13.72	105 ± 18.68	99 ± 17.37	0.183
INR	0.98 ± 5.68	0.97 ± 3.27	1.01 ± 0.10	0.088	0.98 ± 5.75	0.97 ± 3.32	1.005 ± 0.10	0.062
D‐dimer(mg/L)	0.55 ± 0.75	9.62 ± 0.98	1.16 ± 7.46	0.357	0.555 ± 0.75	0.625 ± 0.99	1.045 ± 1.83	0.210
NRS 2002 ≥ 3	75 (53.96%)	203 (78.08%)	23 (100.00%)	0.306	142 (53.28%)	276 (77.20%)	94 (100.00%)	0.283
ASA≥ III	36 (25.90%)	207 (79.62%)	23 (100.00%)	0.494	66 (24.82%)	281 (78.80%)	94 (100.00%)	0.297
CCI								
0–2	122 (87.77%)	84 (32.31%)	0 (0.00%)		235 (88.32%)	120 (33.60%)	0 (0.00%)	
3	15 (10.79%)	77 (29.62%)	0 (0.00%)	0.479	27 (10.22%)	103 (28.80%)	0 (0.00%)	0.439
≥ 4	2 (1.44%)	99 (38.08%)	23 (100.00%)		4 (1.46%)	134 (37.60%)	94 (100.00%)	

Abbreviations: ASA, American Society of Anesthesiologists; BMI, body mass index; CAD, coronary artery disease; CCI, Charlson Comorbidity Index; CKD, chronic kidney disease; CVD, cardiovascular disease; HGB, hemoglobin; INR, international normalized ratio; IPTW, inverse probability of treatment weighting; NRS 2002, nutrition risk screening 2002; PD, classic pancreaticoduodenectomy; PD, pancreaticoduodenectomy, classic; PLT, platelet; PPPD, pylorus‐preserving pancreaticoduodenectomy; PTA, prothrombin activity; Scr, serum creatinine; SMD, standardized mean difference; TB, total bilirubin; WBC, white blood cell.

### 3.2 Comparison of Primary and Secondary Outcomes After IPTW

Table 2 presents a comparison of primary and secondary outcomes after IPTW. Comparing postoperative outcomes, patients aged ≥ 80 years had slightly higher in bile leakage (4.55% vs. 2.00% in the 60–79 years group and 2.19% in the < 60 years group), POPF (13.64% vs. 13.20% in the 60–79 years group and 10.22% in the < 60 years group), other complications (22.73% vs. 14.00% in the 60–79 years group and 14.60% in the < 60 years group), and prolonged postoperative hospitalization (22.73% vs. 15.60% in the 60–79 years group and 13.14% in the < 60 years group). Conversely, they had lower incidences of the rate of DGE (22.73% vs. 22.40% in the 60–79 years group and 29.20% in the < 60 years group) and PPH (9.09% vs. 11.60% in the 60–79 years group and 10.22% in the < 60 years group). All those results had no statistically significant differences (*p* > 0.05). However, the ≥ 80 years group exhibited a significantly higher 90‐day postoperative mortality rate (13.64% vs. 2.00% in the 60–79 years group and 1.46% in the < 60 years group). Age ≥ 80 was significantly associated with 90‐day postoperative mortality (OR: 3.586, 95% CI [1.120, 11.482], *p* = 0.031). The sensitivity analysis results using PSM are consistent with those after IPTW (Table 3). No statistically significant differences were observed between those older than 80 years and other age groups in terms of overall complications, bile leakage, POPF, DGE, PPH, other complications, and prolonged postoperative hospitalization (*p >* 0.05); Age ≥ 80 was significantly associated with 90‐day postoperative mortality (OR: 8.400, 95% CI [2.020, 34.932], *p* = 0.033).

**TABLE 2:** Postoperative Outcomes among patients < 60 years, 60–79 years, and ≥ 80 years after IPTW.

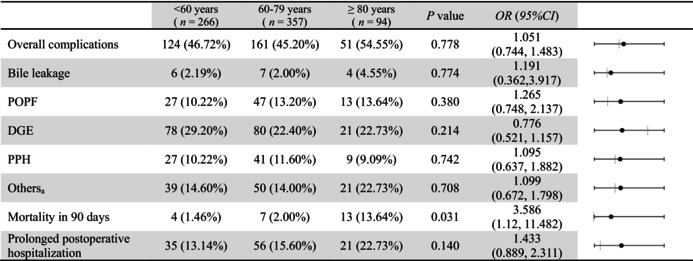

*Note:* a: Including pneumonia, wound or abdominal infections, pleural or abdominal effusion, chylous leakage, acute myocardial infarction and pulmonary embolism.

Abbreviations: 95% CI, 95% confidence interval; DGE, delayed gastric emptying; OR, odds ratio; POPF, postoperative pancreatic fistula; PPH, post pancreatectomy hemorrhage.

**TABLE 3:** Sensitivity analysis by propensity score matching (PSM).

	*p*	OR (95% CI)
Overall complications	0.556	1.288 (0.555, 2.987)
Bile leakage	0.461	0.222 (0.266, 18.559)
POPF	0.914	1.071 (0.307, 3.739)
DGE	0.720	0.831 (0.301, 2.295)
PPH	0.679	1.731 (0.166, 3.219)
Others	0.294	1.737 (0.620, 4.871)
Mortality in 90 days	0.033	8.400 (2.020, 34.932)
Prolonged postoperative hospitalization	0.163	1.994 (0.756, 5.262)

### 3.3 Subgroup Analysis

Age ≥ 80 was identified as a significant risk factor for 90‐day mortality (OR: 8.250, 95% CI [1.984, 34.311], *p* = 0.004). CAD was a significant risk factor for both overall complications (OR: 4.235, 95% CI [1.532, 11.708], *p* = 0.005) and 90‐day mortality (OR: 8.707, 95% CI [2.086, 36.338], *p* = 0.003). Nutritional risk (NRS ≥ 3) and hypertension also contributed to higher complication rates. However, drainage of the pancreatic duct stent was identified as a protective factor for overall complications (OR: 0.469, 95% CI [0.235, 0.935], *p* = 0.031). Subgroup analysis results are detailed in Table 4.

**TABLE 4:** Subgroup analysis of overall complications and mortality in 90 days after surgery by logistic regression.

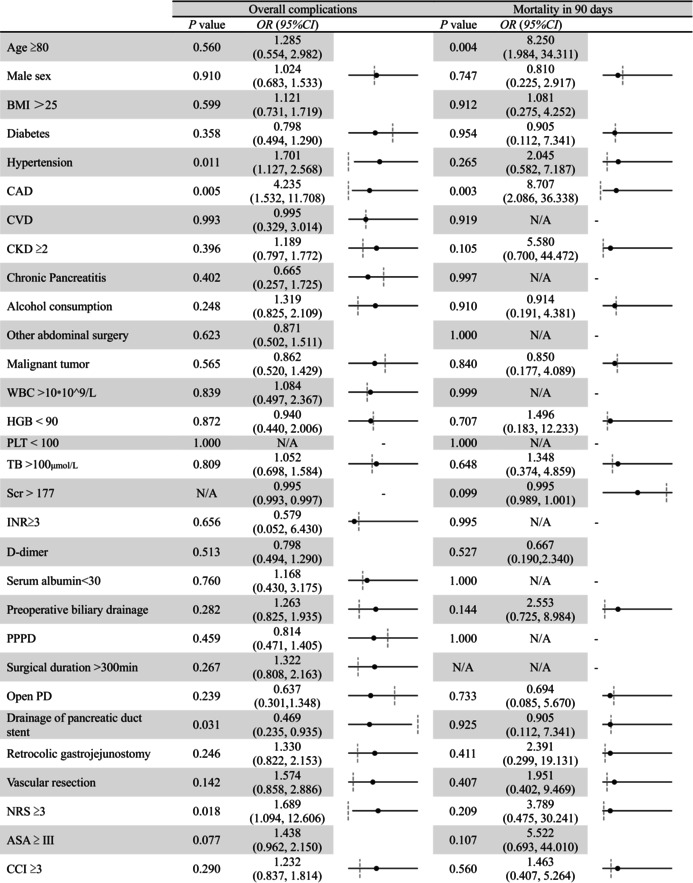

### 3.4 Long‐Term Survival Follow‐Up

**FIGURE 2:**
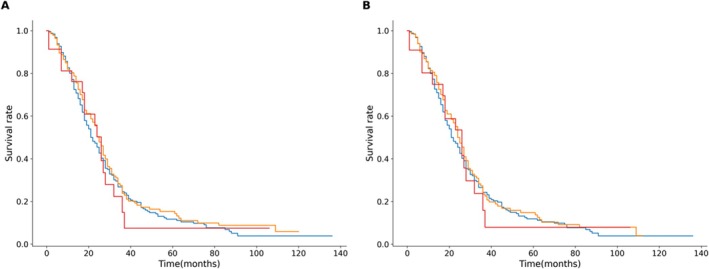
Kaplan–Meier Curve among patients < 60 years, 60–79 years, and ≥ 80 years. (A) Kaplan–Meier survival curve for each age group before IPTW, blue represents the group < 60 years, orange represents the group 60–79 years, red represents the group ≥ 80 years. (B) Kaplan–Meier survival curve for each age group after IPTW, blue represents the group < 60 years, orange represents the group 60–79 years, red represents the group ≥ 80 years.

Median survival times (*MST*) were similar across age groups (Figure 2), with no significant differences observed after IPTW adjustment in Table 5 (MST for < 60 years: 21 months, 60–79 years: 24 months, ≥ 80 years: 26 months, *p* = 0.577).

**TABLE 5:** Median survival times among patients < 60 years, 60–79 years, and ≥ 80 years.

	Before IPTW				After IPTW			
	< 60 years	60–79 years	≥ 80 years	*p*	< 60 years	60–79 years	≥ 80 years	*p*
MST (months)	21	25	26	0.547	21	24	26	0.577

Abbreviation: MST, median survival times.

## 4 Discussion

The growing elderly population presents challenges in the management of complex surgeries, including PD [1, 17]. Elderly patients often face a decline in major organ functions, reduced immune responses, and multiple comorbidities, all of which significantly lower their tolerance to major surgical trauma. This makes the management of such patients particularly challenging [18]. Previous studies, such as that by He et al. [19] have shown that surgical mortality rates in patients aged 70 and above undergoing PD are significantly higher compared to younger cohorts. Univariate analysis showed that the surgical mortality rate of patients aged ≥ 70 years was significantly higher than that of the under‐70 age group. Despite advancements in surgical techniques and perioperative care, which have reduced postoperative mortality rates in major medical centers to below 5%, the incidence of complications following PD remains high [20, 21]. For instance, studies have reported postoperative complication rates as high as 24.4% in patients over 75 years old, with these complications occurring more frequently than in younger patients [22, 23]. A meta‐analysis showed that among patients undergoing PD, those aged 80 and above had a higher 30‐day postoperative mortality rate (OR: 2.22, 95% CI [1.48–3.31], *p* < 0.001) and longer hospital stays (OR: 2.23, 95% CI [1.36–3.10], *p* < 0.001). The overall postoperative complication rate was also higher (OR: 1.51, 95% CI [1.25–1.83], *p* < 0.001), but there was no significant difference in the incidence of POPF and bile leakage [24]. In recent years, some studies have demonstrated that performing PD surgery on elderly patients is safe and feasible. Namur et al. [25] compared the postoperative complication rates of patients over 75 years old and younger undergoing PD, finding no significant difference in the rates of severe complications (17% vs. 10%, *p* = 0.72) or all complications (50% vs. 50%, *p* > 0.05) between the two groups. Lee et al. [26] found no significant differences in overall complications (47% vs. 51%, *p* = 0.54), major complications (19% vs. 25%, *p* = 0.25), and mortality (5% vs. 4%, *p* = 0.53) after PD between elderly patients (≥ 80 years) and younger patients. However, the median survival time was shorter for elderly patients (11.6 months vs. 18.1 months).

Our study applied IPTW to adjust for potential confounding factors, particularly where there was a significant difference in sample sizes among the three age groups. Unlike direct matching, IPTW allows for the retention of data from all study participants, maximizing sample size and increasing statistical power. This method also enhances the validity of causal inferences by making group comparisons in non‐randomized studies more akin to those in randomized trials [27, 28, 29, 30].

Through logistic regression analysis, we identified hypertension and CAD as independent risk factors for postoperative complications. Additionally, CAD was found to be an independent risk factor for 90‐day mortality. Hypertension may contribute to systemic vascular sclerosis and damage, impairing the healing process at the surgical site and increasing the likelihood of POPF and PPH [31, 32]. Patients with CAD may have atherosclerosis in the gastrointestinal arteries, which could elevate the risk of non‐cardiovascular complications following PD [33, 34]. Patients with preoperative cardiovascular diseases should undergo comprehensive systemic medical examinations or multidisciplinary team (MDT) consultations to more thoroughly assess overall organ function, particularly cardiac and pulmonary function [35, 36]. This may help reduce the occurrence of postoperative complications or even mortality, as well as shorten hospital stay duration.

Nutritional risk, as indicated by an NRS score ≥ 3, was also associated with higher complication rates, emphasizing the need for early nutritional intervention. Tumas et al. [37] discovered that nutritional deficiencies had a substantial negative impact on the rates of postoperative complications following PD; another retrospective study also confirmed that an increase in the NRS score was associated with surgical site infection (SSI) after PD [38]. Elevated NRS scores, indicative of malnutrition, impair anastomotic integrity via hypoalbuminemia‐driven collagen deficiency and increase infection risk through lymphocyte dysfunction after PD—a dual mechanism directly linking nutrition to the procedure's most lethal complications [39, 40, 41]. All patients should undergo NRS screening before surgery, with prompt nutritional intervention for deficits, to mitigate these risks.

Drainage of pancreatic duct stent, which can reduce the incidence of postoperative complications, has gradually become a consensus among pancreatic surgeons and is confirmed in our study as well [42, 43, 44, 45]. It is identified as a protective factor and should be considered a routine procedure to reduce the risk of postoperative complications.

A particular concern in our study is the significantly higher 90‐day mortality rate in the age ≥ 80 group. This may be related to the smaller number of patients in the age ≥ 80 group. The causes of death among these patients included hemorrhagic shock due to hepatic artery aneurysm rupture, sudden cardiac death from acute myocardial infarction, and respiratory failure due to lung infection. Although the incidence of postoperative complications was similar to that in younger groups, elderly patients often have more comorbidities and weaker overall organ function, which may contribute to higher mortality rates [46]. Previous studies have shown that comorbidities such as pulmonary dysfunction, hypertension, arrhythmias, and diabetes are more prevalent in elderly patients, increasing the risks during anesthesia and postoperative recovery [2]. Therefore, a thorough preoperative assessment of the patient's overall condition, coupled with rigorous interdisciplinary collaboration, is crucial. Da et al. [47] indicated that patients classified as ASA 3–4 require longer hospital stays and experience longer surgical durations, with significantly increased postoperative mortality and complication rates compared to ASA 1–2 patients. For such patients, strict control of comorbidities, nutritional support, and improvement in organ function should be prioritized before considering surgery. It is also necessary to closely monitor the patient's examination results, changes in vital signs, the amount and color of intra‐abdominal drainage fluid after surgery, and promptly handle any postoperative complications discovered. We think that when assessing the surgical risk of PD in elderly patients, greater attention should be paid to the overall health status of the patients. A comprehensive individualized analysis should be conducted, rather than relying solely on age as a determining factor. However, evidence is still insufficient to draw definitive conclusions, and further randomized controlled trials with larger sample sizes are necessary to verify these findings.

## 5 Limitation

Our study has several limitations. Firstly, as a retrospective study, it is inherently prone to selection bias and information bias. The reliance on existing medical records, which may be incomplete or inaccurate, further compounds these issues. Secondly, the study was conducted at a single institution, which limits the generalizability of our findings. Although the study was conducted by a single surgeon, which eliminates variability in surgical skill levels between different surgeons, differences in patient characteristics and medical practices at other institutions may affect the applicability of our results. Additionally, the small number of cases for certain complications raises concerns about insufficient statistical power. Despite adjusting for known confounders, unmeasured variables could still impact our results. Finally, the long duration of data collection means that advancements in medical technology and treatment strategies over time may have influenced the reliability of our findings. Future prospective multicenter studies with larger patient populations are needed to address these limitations.

## 6 Conclusion

Pancreaticoduodenectomy is a safe and feasible procedure for patients aged 80 and above, offering outcomes comparable to those of younger populations. However, careful preoperative evaluation, especially for cardiovascular and nutritional risks, is crucial to improving postoperative outcomes and minimizing mortality.

## Author Contributions

Conceptualization: Yongliang Sun, Zhiying Yang; Formal analysis, writing review and editing: Tianxiao Wang, Yue Qiu; Data curation: Yingjixing Luo, Ruili Wei, Li Xu, Jia Huang, Wenying Zhou, Hanchun Huang. All authors have read and agreed to the published version of the manuscript.

## Ethics Statement

This is a retrospective single‐institution series analysis study which is clinically conducted at China‐Japan Friendship Hospital. The study adhered to the principles of the Declaration of Helsinki and was authorized by the Ethics Committee of the China‐Japan Friendship Hospital [2024‐KY‐385‐1].

## Conflicts of Interest

The authors declare no conflicts of interest.
